# Supplementary material for: Adversarial and variational autoencoders improve metagenomic binning
Source: Commun Biol. 2023 Oct 21;6:1073. doi: 10.1038/s42003-023-05452-3 (PMC10590447; doi:10.1038/s42003-023-05452-3)
Supplement: Supplementary file 2 — Supplementary Information [file 42003_2023_5452_MOESM2_ESM.docx]

**ADVERSARIAL AND VARIATIONAL AUTOENCODERS IMPROVE METAGENOMIC BINNING**

**AUTHORS**

Pau Piera Líndez^1^, Joachim Johansen^1^, Svetlana Kutuzova^1,2^ ,Arnor Ingi Sigurdsson^1^, Jakob Nybo Nissen^1,*^, Simon Rasmussen^1,3,*^

^1^ Novo Nordisk Foundation Center for Protein Research, Faculty of Health and Medical Sciences, University of Copenhagen, Copenhagen N, 2200, Denmark

^2^ Department of Computer Science, University of Copenhagen, Copenhagen Ø, 2100, Denmark

^3^ The Novo Nordisk Foundation Center for Genomic Mechanisms of Disease, Broad Institute of MIT and Harvard, Cambridge, 02142, USA

* To whom correspondence should be addressed. Email: [jakob.nissen@cpr.ku.dk](mailto:jakob.nissen@cpr.ku.dk), [simon.rasmussen@cpr.ku.dk](mailto:simon.rasmussen@cpr.ku.dk)

**
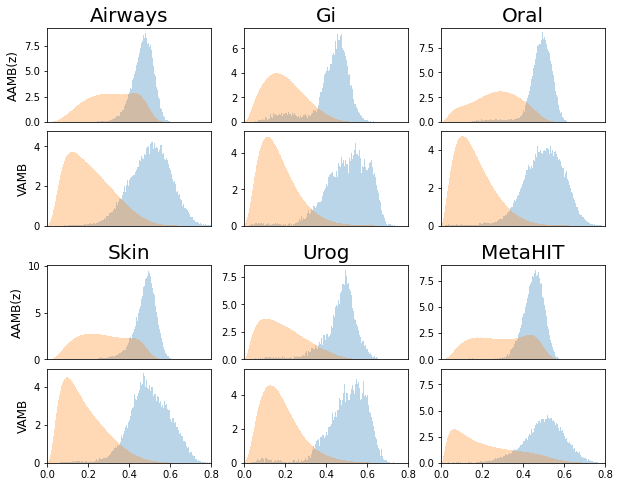
**

Supplementary Figure 1. AAMB(z) and VAMB Intra-inter genome contig distance distributions when trained on CAMI2 and MetaHIT datasets. Distribution of the distances of contigs from the same genome, i.e. intra genome contig distances, are plotted in orange. Distribution of the distances of contigs from different genomes, i.e. inter genome contig distances, are plotted in blue. Distributions are plotted for all CAMI2 and MetaHIT datasets, and for both AAMB(z) and VAMB. Airways, CAMI2 Airways; Gi, CAMI2 Gastrointestinal; Oral, CAMI2 Oral; Skin, CAMI2 Skin; Urog, CAMI2 Urogenital.

**
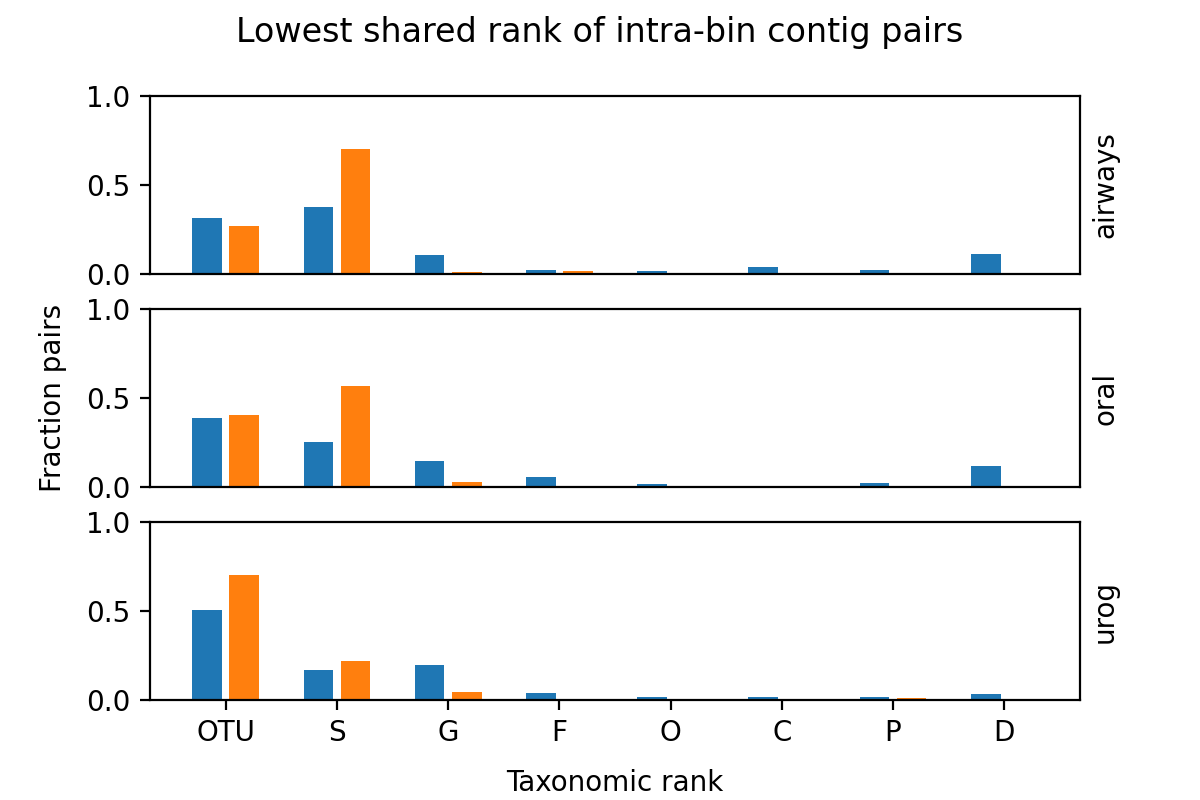
**

Supplementary Figure 2. Phylogenetic distance between contigs from the same AAMB(y) or AAMB(z) cluster. From each cluster in AAMB(y) (blue color) and AAMB(z) (orange color), we sampled 25 contig pairs, and computed the lowest shared taxonomic rank of each pair. The figure shows the distribution among sampled pairs.


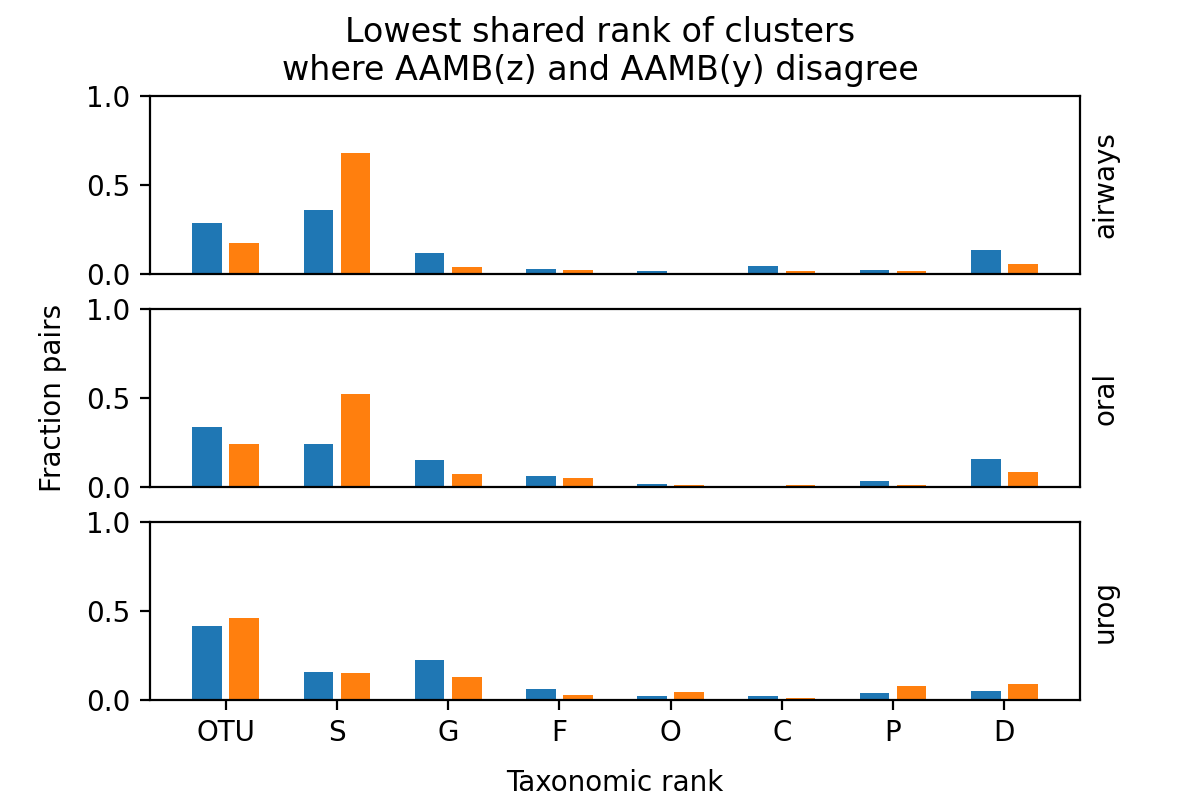


Supplementary Figure 3. Phylogenetic distance between contigs pairs where AAMB(z) and AAMB(y) disagree on whether they belong to the same cluster. Same as Figure 2, but here, within an AAMB cluster, we only sampled pairs of contigs that were placed in different AAMB clusters of the other latent space. Blue bars: Same AAMB(y), different AAMB(z). Orange bars: Same AAMB(z), different AAMB(y).

Supplementary Figure 4. Jaccard correlation indexes between AAMB z, AAMB y and VAMB NC genomes along all benchmark datasets. Jaccard correlation indexes between the NC genomes produced by AAMB *z* latent space, AAMB *y* latent space, VAMB, AAMB z latent space plus AAMB y latent space dereplicated bins, along all benchmark datasets. AAMB(z+y): dereplicated bins from AAMB z and AAMB y latent spaces; Airways, CAMI2 Airways; Gi, CAMI2 Gastrointestinal; Oral, CAMI2 Oral; Skin, CAMI2 Skin; Urog, CAMI2 Urogenital.

Supplementary Figure 5. AAMB VAMB reconstructed genomes analysis and integration for the CAMI2 Gastrointestinal dataset. Comparison of NC strains, species and genus sets of bins generated by VAMB (blue), AAMB(z) (light green), AAMB(y) (dark green), as well as the integrated dereplicated AAMB(z) plus AAMB(y) (light purple), AAMB(z) plus AAMB(y) plus VAMB dereplicated bins (dark purple). Gi, CAMI2 Gastrointestinal.

Supplementary Figure 6. AAMB VAMB reconstructed genomes analysis and integration for the CAMI2 Oral dataset. Comparison of NC strains, species and genus sets of bins generated by VAMB (blue), AAMB(z) (light green), AAMB(y) (dark green), as well as the integrated dereplicated AAMB(z) plus AAMB(y) (light purple), AAMB(z) plus AAMB(*y*) plus VAMB dereplicated bins (dark purple). Oral, CAMI2 Oral.

Supplementary Figure 7. AAMB VAMB reconstructed genomes analysis and integration for the CAMI2 Skin dataset. Comparison of NC strains, species and genus sets of bins generated by VAMB (blue), AAMB(z) (light green), AAMB(y) (dark green), as well as the integrated dereplicated AAMB(z) plius AAMB(y) (light purple), AAMB(z) plus AAMB(y) plus VAMB dereplicated bins (dark purple). Skin, CAMI2 Skin.

Supplementary Figure 8. AAMB VAMB reconstructed genomes analysis and integration for the CAMI2 Urogenital dataset. Comparison of NC strains, species and genus sets of bins generated by VAMB (blue), AAMB(z) (light green), AAMB(y) (dark green), as well as the integrated dereplicated AAMB(z) plus AAMB(y) (light purple), AAMB(z) plus AAMB(y) plus VAMB dereplicated bins (dark purple). Urogenital, CAMI2 Urogenital.

Supplementary Figure 9. AAMB VAMB reconstructed genomes analysis and integration for the MetaHIT dataset. Comparison of NC strains, species and genus sets of bins generated by VAMB (blue), AAMB(z) (light green), AAMB(y) (dark green), as well as the integrated dereplicated AAMB(z) plus AAMB(y) (light purple), AAMB(z) plus AAMB(y) plus VAMB dereplicated bins (dark purple).


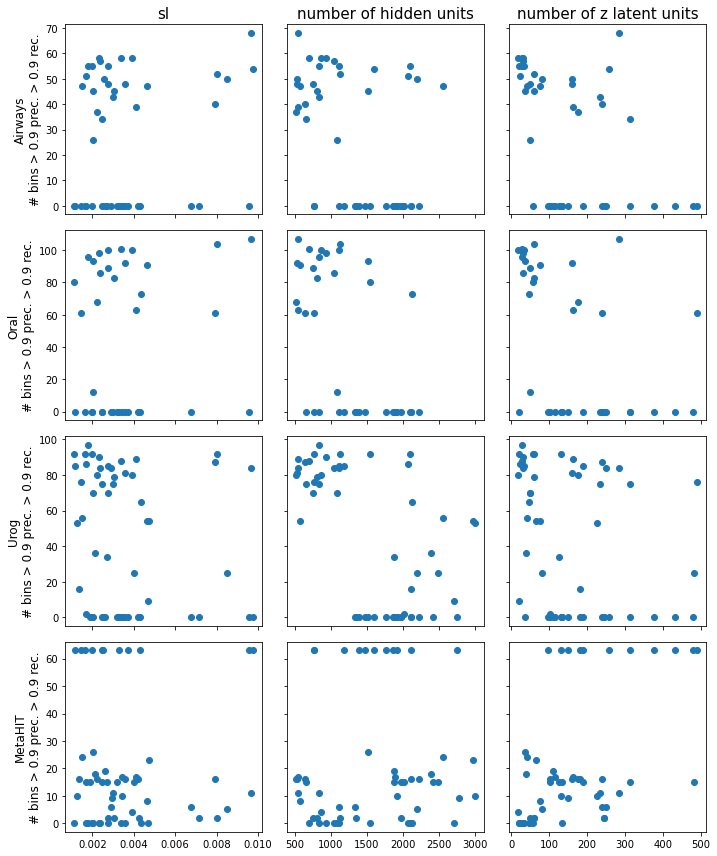


Supplementary Figure 10. First round of hyperparameter random searches. AAMB(z) bins above 0.9 precision and 0.9 recall are shown for Airways, Oral and Urogenital (Urog) CAMI2 datasets as well as for the MetaHIT dataset. Hyperparmeters evaluated are the loss scale (sl), the number of hidden units of the encoder-decoder (number of hidden units) and the number of latent units of the *z* latent space (number of latent units).


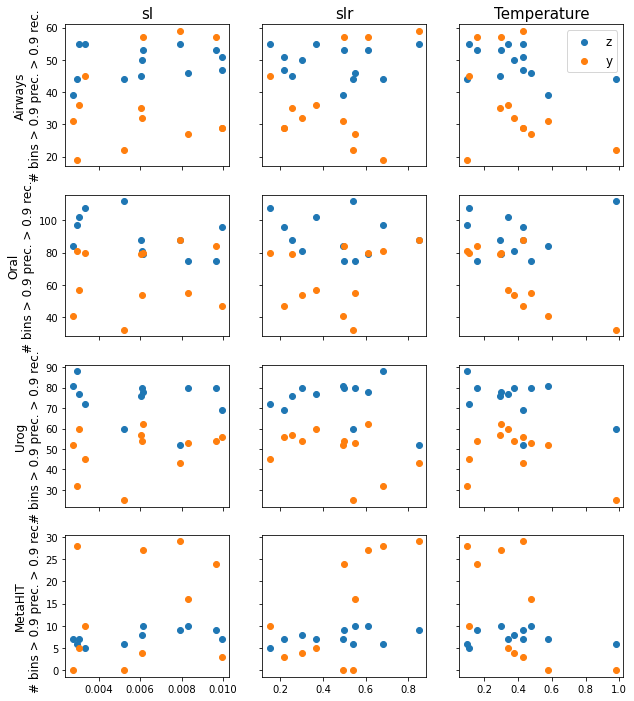


Supplementary Figure 11. Second round of hyperparameter random searches. AAMB(z), AAMB(y) bins above 0.9 precision and 0.9 recall are shown for Airways, Oral and Urogenital (Urog) CAMI2 datasets as well as for the MetaHIT dataset. Hyperparameters evaluated are the loss scale (sl), the discriminator loss scale (sl), and the Temperature parameter from the RelaxedOneHotCategorical distribution (Temperature). *z* , NC genomes reconstructed by the *z* latent space; *y*, NC genomes reconstructed by the *y* latent space.

Supplementary Figure 12. Dereplication process after running AAMB and VAMB. **A**. Completeness and contamination of AAMB(z) and VAMB bins is estimated with CheckM2, allowing the selection of bins with contamination below 5% and completeness above 90% (NC bins). During the dereplication step, NC bins from different binners are pairwise compared by contigs composition, defining a pair of bins “near-identical” if such bins align with 100% identity over at least 75% coverage of the smallest bin. For each “near-identical” pair of bins, the bin with the highest score is selected (see Methods). **B**. If some contigs are present in two bins after the dereplication step, two new bins are created without the intersecting contigs, named “ripped bins”. Subsequently, completeness and contamination scores are estimated over the “ripped bins” with CheckM2. The intersecting contigs are assigned to the ripped bin that has the largest decrease in score with respect the original bin, whereas the ripped bin with the smaller decrease in score loses the intersecting contig.


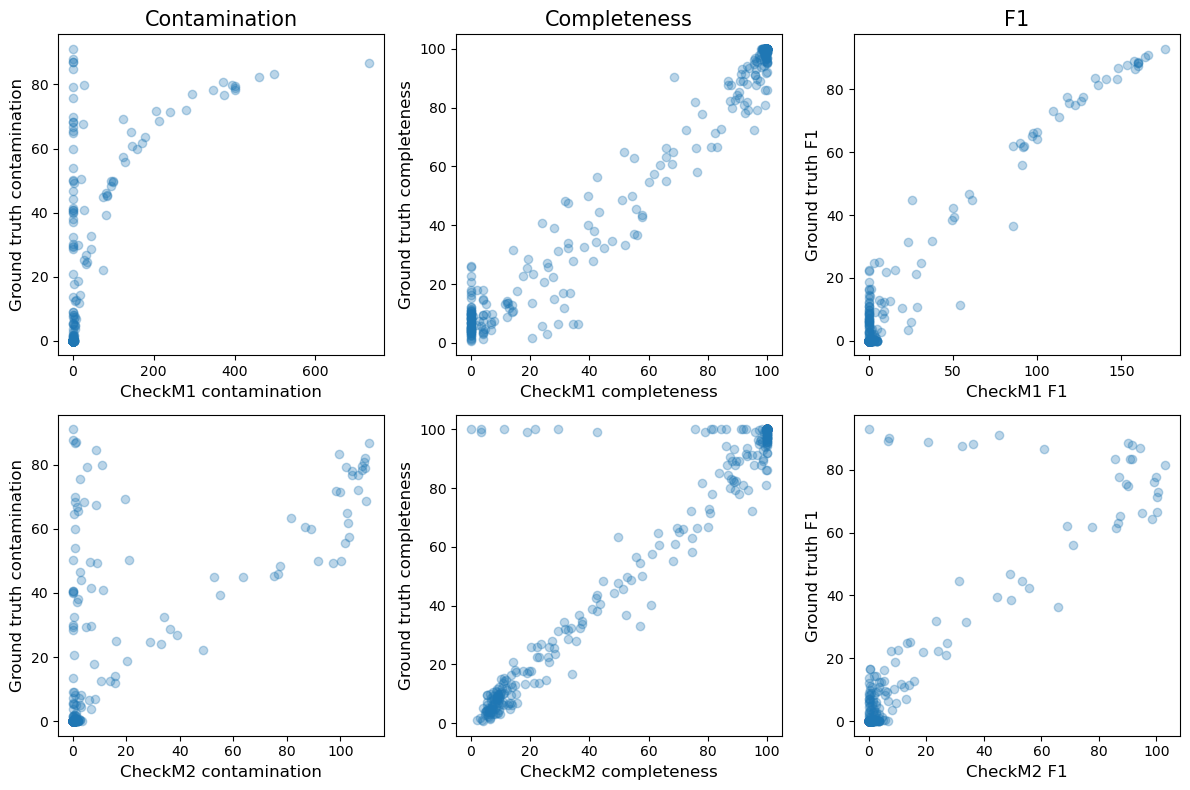


Supplementary Figure 13. CheckM1 and CheckM2 contamination, completeness, and F1 with respect the CAMI Airways ground truth contamination, completeness, and F1. Bins reconstructed by AAMB(z) executed on the CAMI Airways dataset. For each bin, completeness and contamination were obtained with CheckM1, CheckM2 and the gold standard reference.

| Dataset | AAMB(y) | AAMB(z) | Dereplicated | Lost |
| --- | --- | --- | --- | --- |
| Airways | 34 | 69 | 74 | 7 |
| GI | 65 | 94 | 98 | 5 |
| Oral | 69 | 105 | 118 | 6 |
| Skin | 44 | 81 | 90 | 2 |
| Urog | 31 | 56 | 58 | 3 |
| MetaHIT | 29 | 11 | 22 | 7 |
| Total | 272 | 416 | 460 | 30 |

Supplementary Table 1. dRep dereplication efficiency for AAMB near complete genomes. Efficiency of the dereplication pipeline to dereplicate and integrate near complete genomes from AAMB(z) and AAMB(y). AAMB(y): Near complete genomes reconstructed from the y AAMB latent space. AAMB(z): Near complete genomes reconstructed from the z AAMB latent space. Dereplicated: dereplication pipeline output from the AAMB(z) and AAMB(y) NC genomes. Lost: AAMB(z) or AAMB(y) near complete genomes lost during the dereplication process. Airways: CAMI2 Airways, Oral: CAMI2 Oral, Urog: CAMI2 Urogenital, GI: CAMI2 Gastrointestinal, Skin: CAMI2 Skin.

| Dataset | AAMB(z) | AAMB(z) Y split |
| --- | --- | --- |
| Airways | 69 | 70 |
| Gi | 94 | 97 |
| Oral | 105 | 101 |
| Skin | 81 | 82 |
| Urog | 56 | 71 |

Supplementary Table 2. NC bins reconstructed from AAMB(z) and AAMB(z) split by their AAMB(y) label. If our initial hypothesis that the y vector captures high-level variance and the z vector captures low-level variance, then some z clusters could be composite clusters of different y, i.e. distant organisms with similar low-level variance. However, splitting the z clusters by their y-label does not improve performance. We did hyperparameter optimisation to improve AAMB(z) y split performance but could not get it better than AAMB(z) (data not shown). In total, this goes against our initial hypothesis.

| AAMB(z) + AAMB(y) | | | | | |
| --- | --- | --- | --- | --- | --- |
| Dataset | Total | dRep_CheckM2 | dRep_CheckM2 + ripping | manual dereplication | manual dereplication + ripping |
| Airways | 81 | 71 | 71 | 74 | 74 |
| Gi | 103 | 98 | 99 | 98 | 98 |
| Oral | 124 | 118 | 117 | 119 | 118 |
| Skin | 92 | 89 | 89 | 90 | 90 |
| Urog | 71 | 69 | 69 | 70 | 70 |
| MetaHIT | 29 | 15 | 15 | 22 | 22 |
| Almeida | N/A | 5078 | 5071 | 5078 | 5077 |
| HMP2 | N/A | 2723 | 2716 | 2723 | 2715 |
| AAMB(z) + AAMB(y)+VAMB | | | | | |
| Airways | 87 | 77 | 77 | 82 | 82 |
| Gi | 109 | 103 | 104 | 103 | 103 |
| Oral | 145 | 138 | 137 | 140 | 138 |
| Skin | 106 | 103 | 103 | 104 | 104 |
| Urog | 86 | 81 | 81 | 83 | 83 |
| MetaHIT | 29 | 15 | 15 | 22 | 22 |
| Almeida | N/A | 5748 | 5733 | 5748 | 5733 |
| HMP2 | N/A | 3579 | 3568 | 3573 | 3569 |

Supplementary Table 3. Dereplication efficiency for AAMB and AVAMB near complete bins applying different dereplication strategies. Dereplication efficiency evaluated for dRep, dRep and ripping, manual dereplication, manual dereplication and ripping. Dereplication strategies were applied to deduplicate and integrate near complete bins from AAMB y and z latent spaces, and from AAMB z, y latent spaces and VAMB. dRep: dereplication obtained by running dRep per sample. dRep + ripping: dereplication obtained by running dRep per sample and bins ripping applied subsequently. Manual dereplication: manual dereplication. Manual dereplication + ripping: manual dereplication and bins ripping applied subsequently.

| AAMB(z) + AAMB(y) | | | | |
| --- | --- | --- | --- | --- |
| dataset | dRep | dRep + ripping | Manual dereplication | Manual dereplication + ripping |
| Airways | 0 | 0 | 0 | 0 |
| Gi | 3 | 0 | 3 | 0 |
| Oral | 3 | 0 | 3 | 0 |
| Skin | 0 | 0 | 0 | 0 |
| Urog | 0 | 0 | 0 | 0 |
| MetaHIT | 0 | 0 | 0 | 0 |
| Almeida | 129 | 0 | 129 | 0 |
| HMP2 | 66 | 0 | 66 | 0 |
| AAMB(z) + AAMB(y) + VAMB | | | | |
| Airways | 0 | 0 | 0 | 0 |
| Gi | 18 | 0 | 18 | 0 |
| Oral | 7 | 0 | 6 | 0 |
| Skin | 0 | 0 | 0 | 0 |
| Urog | 2 | 0 | 2 | 0 |
| MetaHIT | 0 | 0 | 0 | 0 |
| Almeida | 280 | 0 | 285 | 0 |
| HMP2 | 229 | 0 | 228 | 0 |

Supplementary Table 4. Remaining duplicated contigs after applying different dereplication strategies to AAMB and AVAMB near complete bins. Remaining duplicated contigs after dereplicating NC bins from AAMB and AVAMB with dRep, dRep and ripping, manual dereplication, manual dereplication and ripping. Dereplication strategies were applied to deduplicate near complete bins from AAMB z and y latent spaces and from AAMB z , y latent spaces and VAMB.. dRep: dereplication obtained by running dRep per sample. dRep + ripping: dereplication obtained by running dRep per sample and bins ripping applied subsequently. Manual dereplication: manual dereplication. Manual dereplication + ripping: manual dereplication and bins ripping applied subsequently.

|  |  | CPU | | | | GPU | | | |  |  |
| --- | --- | --- | --- | --- | --- | --- | --- | --- | --- | --- | --- |
| Dataset | BAM files  parse | VAMB | | AAMB | | VAMB | | AAMB | | CheckM2 | Dereplicate |
|  |  | Train | Cluster | Train | Cluster | Train | Cluster | Train | Cluster |  |  |
| Airways | 3.3 | 27.4 | 2.1 | 35.7 | 34.5 | 6.8 | 1.2 | 10.7 | 4.8 | 210(40.6) | 2.5 |
| Gi | 4.8 | 12.2 | 0.3 | 16.5 | 0.9 | 3.35 | 0.4 | 4.7 | 0.5 | 244(48.1) | 1 |
| Oral | 4.5 | 15 | 2.5 | 38.5 | 38.3 | 7.3 | 1.2 | 11.5 | 6.4 | 250(48.5) | 2 |
| Skin | 3.8 | 26 | 1.5 | 33.4 | 23.7 | 6.6 | 1 | 10.1 | 2.4 | 242(46.3) | 2 |
| Urog | 3 | 8.9 | 0.3 | 11.2 | 0.9 | 2.8 | 0.4 | 3.4 | 0.4 | 159(32.5) | 0 |

Supplementary Table 5. AVAMB running times both with and without GPU for all stages of the process. The BAM files parse column indicates the contigs depths estimation runtime in minutes implemented on VAMB. AAMB, VAMB train and cluster columns represent the running times in minutes when running those models on the CAMI2 datasets with 16 CPU threads and 20 gigabytes with and without GPU. After clustering, AAMB/VAMB clusters were evaluated with Checkm2. Checkm2 was run with 8 threads and 8 Gb of memory per sample. Checkm2 runtime is expressed for all samples (average runtime per sample in minutes). Dereplication shows the runtime in minutes required to dereplicate the NC genomes generated. All stages (bam files parsing, training, clustering, bins evaluation with checkm2 and NC bins dereplication) were integrated with snakemake for convenience and parallelization purposes. Airways: CAMI2 Airways, Oral: CAMI2 Oral, Urog: CAMI2 Urogenital, GI: CAMI2 Gastrointestinal, Skin: CAMI2 Skin.

| Dataset | AAMB(y) | AAMB(z) | VAMB | Dereplicated | Lost |
| --- | --- | --- | --- | --- | --- |
| Airways | 34 | 69 | 63 | 82 | 5 |
| GI | 65 | 94 | 86 | 103 | 6 |
| Oral | 69 | 105 | 124 | 138 | 6 |
| Skin | 44 | 81 | 77 | 104 | 2 |
| Urog | 31 | 56 | 78 | 83 | 4 |
| MetaHIT | 29 | 11 | 17 | 22 | 7 |
| Total | 272 | 416 | 445 | 532 | 30 |

Supplementary Table 6. Dereplication efficiency for AAMB and VAMB near complete genomes. Efficiency of the dereplication pipeline to dereplicate and integrate near complete genomes from AAMB(y), AAMB(z) and VAMB. AAMB(y): Near complete genomes reconstructed from the *y* AAMB latent space. AAMB(z): Near complete genomes reconstructed from the *z* AAMB latent space. VAMB: Near complete genomes reconstructed by VAMB. Dereplicated: dereplication pipeline output from the AAMB(z), AAMB(y) and VAMB NC genomes. Lost: AAMB(z), AAMB(y) or VAMB near complete genomes lost during the dereplication process. Airways: CAMI2 Airways, Oral: CAMI2 Oral, Urog: CAMI2 Urogenital, GI: CAMI2 Gastrointestinal, Skin: CAMI2 Skin.

| Dataset/model set | AAMB(z) unique | AAMB(y) unique | VAMB unique | AAMB(z) and AAMB(y) | AAMB(z) and VAMB | AAMB(y) and VAMB | AAMB(y) and AAMB(z) and VAMB | All |
| --- | --- | --- | --- | --- | --- | --- | --- | --- |
| Airways | 14 | 3 | 6 | 3 | 31 | 6 | 19 | 82 |
| Gi | 13 | 3 | 5 | 4 | 22 | 5 | 51 | 103 |
| Oral | 12 | 5 | 18 | 2 | 41 | 14 | 47 | 139 |
| Skin | 22 | 2 | 14 | 3 | 25 | 8 | 30 | 104 |
| Urog | 5 | 3 | 13 | 0 | 33 | 1 | 28 | 83 |
| MetaHIT | 0 | 6 | 0 | 1 | 0 | 6 | 9 | 22 |

Supplementary Table 7. VAMB AAMB NC genomes contributions and intersections. AAMB and VAMB specific contributions and intersections on CAMI2 and MetaHIT datasets. Unique stands for NC genomes only reconstructed by the given model. And stands for NC genomes reconstructed by models and not any of the rest. All presents the addition of all sets which at the same time expresses the total amount of NC genomes reconstructed by VAMB and AAMB when dereplicating with dRep. Airways: CAMI2 Airways, Oral: CAMI2 Oral, Urog: CAMI2 Urogenital, GI: CAMI2 Gastrointestinal, Skin: CAMI2 Skin. NC: Near complete.

| Dataset | A | V | M | V+A | V+M | A+M | V+M+A |
| --- | --- | --- | --- | --- | --- | --- | --- |
| Airways | 74 | 63 | 36 | 82 | 61 | 77 | 84 |
| GI | 98 | 86 | 76 | 103 | 101 | 108 | 112 |
| Oral | 118 | 124 | 68 | 138 | 129 | 128 | 145 |
| Skin | 90 | 72 | 62 | 104 | 97 | 98 | 111 |
| Urog | 70 | 78 | 66 | 83 | 88 | 88 | 96 |
| MetaHIT | 22 | 17 | 1 | 22 | 13 | 21 | 22 |
| Total | 472 | 440 | 309 | 532 | 489 | 520 | 570 |

Supplementary Table 8. NC genomes reconstructed from the benchmark datasets by AAMB, VAMB, MetaBAT2 individually and when integrating with the dereplication pipeline. Near complete genomes reconstructed by AAMB, VAMB, MetaBAT2, VAMB and AAMB, VAMB and MetaBAT2, AAMB and MetaBAT2, VAMB and AAMB and MetaBAT2. Integration of binning results was done with the dereplication pipeline. A: AAMB, V: VAMB, M: MetaBAT2, V+A: VAMB and AAMB, V+M: VAMB and MetaBAT2, A+M: AAMB +MetaBAT2, V+A+M: VAMB and AAMB and MetaBAT2.

| Dataset | AAMB | VAMB | MetaBAT2 | AVAMB | Semibin | Semibin2 | MetaDecoder |
| --- | --- | --- | --- | --- | --- | --- | --- |
| Airways | 74 | 63 | 36 | 82 | 96 | 94 | 94 |
| GI | 98 | 86 | 76 | 103 | 140 | 149 | 127 |
| Oral | 118 | 124 | 68 | 138 | 159 | 170 | 140 |
| Skin | 90 | 72 | 62 | 104 | 138 | 141 | 133 |
| Urog | 70 | 78 | 66 | 83 | 112 | 118 | 104 |
| Total | 472 | 440 | 309 | 532 | 645 | 672 | 598 |

Supplementary Table 9. Near complete genomes reconstructed from the CAMI2 datasets by AAMB, VAMB, MetaBAT2, AVAMB, SemiBin, SemiBin2, and MetaDecoder.

| Dataset | Airways | GI | Oral | Skin | Urog |
| --- | --- | --- | --- | --- | --- |
| SemiBin | 18.7 | 13.2 | 20.01 | 14.05 | 9.6 |
| SemiBin2 | 6.5 | 6.7 | 7.2 | 6.5 | 4.4 |

Supplementary Table 10. SemiBin and SemiBin2 running times on GPU for the CAMI datasets. SemiBin and SemiBin2 running times in hours when running on 20 CPU threads and 150 gigabytes with one GPU on the CAMI benchmark datasets. GI; Gastrointestinal, Urog; Urogenital.

| Dataset | Airways | GI | Oral | Skin | Urog |
| --- | --- | --- | --- | --- | --- |
| Runtime | 61 | 40 | 84 | 58 | 24 |

Supplementary Table 11. MetaDecoder running times on CPU for the CAMI datasets. MetaDecoder running times in minutes when running on 20 CPU threads and 60 gigabytes on the CAMI benchmark datasets. GI; Gastrointestinal, Urog; Urogenital.

| Dataset | AVAMB | SemiBin2 - no RC |
| --- | --- | --- |
| Airways | 82 | 62 |
| Gi | 103 | 103 |
| Oral | 138 | 130 |
| Skin | 104 | 67 |
| Urog | 83 | 73 |
| Total | 510 | 435 |

Supplementary Table 12. Near complete genomes reconstructed from the CAMI2 datasets by AVAMB and SemiBin2 when SemiBin2 single-copy genes-based re-clustering is not applied on SemiBin2 workflow. GI; Gastrointestinal, Urog; Urogenital; SemiBin2 – no RC: SemiBin2 NC genomes without single-copy genes-based re-clustering.

| Dataset | AVAMB+RC | SemiBin2 | MetaDecoder |
| --- | --- | --- | --- |
| Airways | 102 | 96 | 94 |
| Gi | 145 | 140 | 127 |
| Oral | 165 | 159 | 140 |
| Skin | 143 | 138 | 133 |
| Urog | 111 | 112 | 104 |
| Total | 666 | 645 | 598 |

Supplementary Table 13. Near complete genomes reconstructed from the CAMI2 datasets by AVAMB bins re-clustered with SemiBin2 single-copy genes-based re-clustering implementation, SemiBin2 and MetaDecoder. GI; Gastrointestinal, Urog; Urogenital. AVAMB + RC, AVAMB NC genomes when the single-copy genes-based re-clustering from SemiBin2 was applied to AVAMB’s workflow.

| Dataset | AAMB(z) | AAMB(y) | VAMB | AAMB(z+y) | AAMB+VAMB |
| --- | --- | --- | --- | --- | --- |
| Almeida | 4,355 | 2,891 | 4,630 | 5,077 | 5,733 |
| HMP2 | 2,586 | 623 | 2,655 | 2,715 | 3,569 |

Supplementary Table 14. NC bins reconstructed from the Almeida and Human Microbiome Project 2 datasets by AAMB(z), AAMB(y), VAMB, individually and when integrated. Near complete genomes reconstructed by AAMB(z), AAMB(y), VAMB, AAMB(z) and AAMB(y), VAMB and AAMB(z) and AAMB(y). Integration of binning results was done with the dereplication pipeline. AAMB(z): NC bins reconstructed from AAMB z latent space, AAMB(y): NC bins reconstructed from AAMB y latent space, AAMB(z+y): NC bins reconstructed from AAMB z latent space and AAMB y latent space, AAMB + VAMB: NC bins reconstructed from AAMB z latent space and AAMB y latent space and VAMB.

| Dataset | Parse bam files | VAMB | | AAMB | | CheckM2 | dereplicate |
| --- | --- | --- | --- | --- | --- | --- | --- |
|  |  | Train | Cluster | Train | Cluster |  |  |
| Almeida | 1.4 | 5.32 | 1.6 | 7.22 | 6.8 | 0.25(0.07) | 0.6 |

Supplementary Table 15. AVAMB running times when running with GPU on a high-performance computing station for the Almeida dataset. The BAM files parse column indicates the contigs depths estimation runtime in hours implemented on VAMB. AAMB, VAMB train and cluster columns represent the running times in hours when running those models on the Almeida datasets with 20 CPU threads, 1 GPU, and 100 GB RAM. After clustering, AAMB/VAMB clusters were evaluated with Checkm2. Checkm2 was run on parallel per sample, assigning 25 threads and 40 Gb of memory per sample run. Checkm2 runtime expressed the time spent for all samples (average runtime per sample in hours). Dereplication shows the runtime in hours required to dereplicate the NC genomes generated. All stages (bam files parsing, training, clustering, bins evaluation with Checkm2 and NC bins dereplication) were integrated with snakemake for convenience and parallelization purposes.

| Level | Common | VAMB only | AAMB only | AVAMB |
| --- | --- | --- | --- | --- |
| Domain | 2 | 0 | 0 | 2 |
| Phylum | 13 | 0 | 0 | 13 |
| Class | 16 | 0 | 0 | 16 |
| Order | 38 | 2 | 1 | 41 |
| Family | 81 | 5 | 3 | 89 |
| Genus | 286 | 22 | 18 | 325 |
| Species | 614 | 63 | 93 | 767 |

Supplementary Table 16. We annotated all Almeida NC bins dereplicated bins from AAMB and VAMB with GTDB-Tk and counted a particular taxon if at least one genome was reconstructed. Common column presents the amount of taxons with at least one NC bin generated both by VAMB and AAMB, the VAMB only column presents the amount of taxons with at least one NC bin generated only by VAMB, and the AAMB only column presents the amount of taxons with at least one NC bin generated only by AAMB. Worth to mention that the quantities do not reflect how many NC bins were reconstructed for each taxon. Results also represented in Figure 3b.

| Level | Common | VAMB only | AAMB only | AVAMB |
| --- | --- | --- | --- | --- |
| Domain | 2 | 0 | 0 | 2 |
| Phylum | 12 | 0 | 0 | 12 |
| Class | 14 | 0 | 0 | 14 |
| Order | 27 | 1 | 0 | 28 |
| Family | 47 | 3 | 1 | 51 |
| Genus | 122 | 8 | 18 | 148 |
| Species | 200 | 17 | 61 | 278 |

Supplementary Table 17. HMP2 taxons with at least one NC dereplicated bin. We annotated all Human Microbiome Project 2 NC dereplicated bins from AAMB and VAMB with GTDB-Tk and counted a particular taxon if at least one genome was reconstructed. Common column presents the amount of taxons with at least one NC bin generated both by VAMB and AAMB, the VAMB only column presents the amount of taxons with at least one NC bin generated only by VAMB, and the AAMB only column presents the amount of taxons with at least one NC bin generated only by AAMB. Mind that quantities do not reflect how many NC bins were reconstructed for each taxon. Results also represented in Figure 3c.

| Dataset | V = A | V > A | A > V | V unique | A unique | AVAMB |
| --- | --- | --- | --- | --- | --- | --- |
| Almeida | 332 | 1530 | 2091 | 670 | 1110 | 5733 |
| HMP2 | 62 | 961 | 782 | 848 | 916 | 3569 |

Supplementary Table 18. VAMB and AAMB bins from the same sample with 100% identity over at least 75% of the smallest bin. NC: Near complete. V = A: VAMB and AAMB NC bins with exact same score. V > A: VAMB NC bins with higher score than AAMB NC bins, A > V: AAMB NC bins with higher score than VAMB NC bins, V uniq: VAMB NC bins not reconstructed by any AAMB NC bin at the selected identity settings, A unique: AAMB NC bins not reconstructed by any VAMB NC bin at the selected identity settings.

| Level | VAMB | AAMB | AVAMB | MetaBAT2 |
| --- | --- | --- | --- | --- |
| Domain | 2 | 2 | 2 | 1 |
| Phylum | 13 | 13 | 13 | 13 |
| Class | 16 | 16 | 16 | 17 |
| Order | 40 | 39 | 41 | 38 |
| Family | 86 | 84 | 89 | 84 |
| Genus | 308 | 304 | 325 | 285 |
| Species | 677 | 707 | 767 | 605 |

Supplementary Table 19. We annotated all Almeida NC bins dereplicated bins from VAMB, AAMB, AVAMB and MetaBAT2 with GTDB-Tk and counted a particular taxon if at least one genome was reconstructed.

| Level | AVAMB | AVAMB+RC | MetaDecoder |
| --- | --- | --- | --- |
| Domain | 2 | 2 | 1 |
| Phylum | 9 | 9 | 7 |
| Class | 10 | 10 | 8 |
| Order | 17 | 17 | 14 |
| Family | 32 | 32 | 27 |
| Genus | 69 | 69 | 55 |
| Species | 102 | 102 | 69 |

Supplementary Table 20. We annotated all Almeida 30 samples NC bins AVAMB, AVAMB bins re-clustered with SemiBin2 single-copy genes-based re-clustering implementation and MetaDecoder with GTDB-Tk and counted a particular taxon if at least one genome was reconstructed. AVAMB + RC; AVAMB bins re-clustered with SemiBin2 single-copy genes-based re-clustering implementation.

| Dataset | AAMB(z) | AAMB(y) | VAMB | AAMB(z+y) | AAMB + VAMB |
| --- | --- | --- | --- | --- | --- |
| Almeida | 4777 | 3221 | 5070 | 4751 | 5165 |
| HMP2 | 3193 | 703 | 3122 | 2364 | 3095 |

Supplementary Table 21. CheckM NC bins reconstructed from the Almeida and Human Microbiome Project 2 datasets by AAMB(z), AAMB(y), VAMB, AAMB and AVAMB. Near complete genomes according to CheckM reconstructed by AAMB(z), AAMB(y), VAMB, AAMB(z) and AAMB(y), VAMB and AAMB(z) and AAMB(y). Integration of binning results was done with the dereplication pipeline using CheckM2. AAMB(z): NC bins reconstructed from AAMB z latent space, AAMB(y): NC bins reconstructed from AAMB y latent space, AAMB(z+y): NC bins reconstructed from AAMB z latent space and AAMB y latent space, AAMB + VAMB: NC bins reconstructed from AAMB z latent space and AAMB y latent space and VAMB.
