# Supplementary material for: Adversarial and variational autoencoders improve metagenomic binning
Source: Commun Biol. 2023 Oct 21;6:1073. doi: 10.1038/s42003-023-05452-3 (PMC10590447; doi:10.1038/s42003-023-05452-3)
Supplement: Supplementary file 5 — Reporting Summary [file 42003_2023_5452_MOESM5_ESM.pdf]

nature portfolio

Corresponding author(s): Simon Rasmussen

Last updated by author(s): Oct 2, 2023

Reporting Summary

Nature Portfolio wishes to improve the reproducibility of the work that we publish. This form provides structure for consistency and transparency in reporting. For further information on Nature Portfolio policies, see our Editorial Policies and the Editorial Policy Checklist.

Statistics

For all statistical analyses, confirm that the following items are present in the figure legend, table legend, main text, or Methods section.

n/a	Confirmed	
☐	☒	The exact sample size (*n*) for each experimental group/condition, given as a discrete number and unit of measurement
☐	☒	A statement on whether measurements were taken from distinct samples or whether the same sample was measured repeatedly
☒	☐	The statistical test(s) used AND whether they are one- or two-sided<br>*Only common tests should be described solely by name; describe more complex techniques in the Methods section.*
☒	☐	A description of all covariates tested
☒	☐	A description of any assumptions or corrections, such as tests of normality and adjustment for multiple comparisons
☐	☒	A full description of the statistical parameters including central tendency (e.g. means) or other basic estimates (e.g. regression coefficient) AND variation (e.g. standard deviation) or associated estimates of uncertainty (e.g. confidence intervals)
☒	☐	For null hypothesis testing, the test statistic (e.g. *F*, *t*, *r*) with confidence intervals, effect sizes, degrees of freedom and *P* value noted<br>*Give P values as exact values whenever suitable.*
☒	☐	For Bayesian analysis, information on the choice of priors and Markov chain Monte Carlo settings
☒	☐	For hierarchical and complex designs, identification of the appropriate level for tests and full reporting of outcomes
☒	☐	Estimates of effect sizes (e.g. Cohen's *d*, Pearson's *r*), indicating how they were calculated

Our web collection on statistics for biologists contains articles on many of the points above.

Software and code

Policy information about availability of computer code

Data collection	No data was simulated or collected for this study. All datasets used for this study are publicly available (see Data section).
Data analysis	Python 3.9.16<br>CheckM2 v0.1.3<br>PyTorch v.1.7.1<br>CUDA v.8.0.61<br>MetaBAT2 v.2.10.2<br>Minimap2 v.2.15r905<br>GTDB-tk v.2.1.0<br>IQ-TREE v1.6.8.<br>iTOL v.6.7.4<br>dRep v3.0.0<br>Snakemake v.7.22.0<br>SemiBin v0.7.0<br>VAMB v3.0.8<br>SemiBin2 v1.5.1<br>MetaDecoder v1.0.17<br>CheckM v1.2.2

For manuscripts utilizing custom algorithms or software that are central to the research but not yet described in published literature, software must be made available to editors and reviewers. We strongly encourage code deposition in a community repository (e.g. GitHub). See the Nature Portfolio guidelines for submitting code & software for further information.

Data

Policy information about availability of data

All manuscripts must include a data availability statement. This statement should provide the following information, where applicable:

- Accession codes, unique identifiers, or web links for publicly available datasets
- A description of any restrictions on data availability
- For clinical datasets or third party data, please ensure that the statement adheres to our policy

The sequence data used in this study is publicly available, from either the respective studies or from the European Nucleotide Archive (ENA). The semi-synthetic MetaHIT dataset was downloaded from https://portal.nersc.gov/dna/RD/Metagenome_RD/MetaBAT/Files/ as the files depth.txt.gz and assembly-filtered.fa.gz. The simulated CAMI2 datasets were downloaded from https://data.cami-challenge.org/participate from the “2nd CAMI Toy Human Microbiome Project Dataset”, respectively. The Almeida de novo assemblies were downloaded from http://ftp.ebi.ac.uk/pub/databases/metagenomics/umgs_analyses/benchmarked_assemblies.tar.gz and the reads were downloaded from ENA as specified in their publication. The Human Microbiome Project 2 data was originally obtained from the European Nucleotide Archive accession PRJNA398089.

Research involving human participants, their data, or biological material

Policy information about studies with human participants or human data. See also policy information about sex, gender (identity/presentation), and sexual orientation and race, ethnicity and racism.

Reporting on sex and gender	No data was collected from individuals for this study. No data is shared at the individual-level. This study used publicly available data generated by previous studies (see Data section), where to our knowledge, no gender-based or sex-based analysis were performed, nor gender/sex information was provided.
Reporting on race, ethnicity, or other socially relevant groupings	No socially constructed or socially relevant categorization variables were used in this study. No controlling for confounding variables was performed in this study since confounding effects have no impact on implementation of the method developed in this study.
Population characteristics	No human research participants were recruited for this study. However, the data generated by the Human Microbiome Project 2 used in this study, originated from the Inflammatory Bowel Disease cohort consisting of 1,338 samples from 121 a total of 27 healthy controls, 65 Crohn's Disease, and 38 Ulcerative Colitis patients from Lloyd-Price et al. To our knowledge, no covariate-relevant population information is provided for the remaining data used in this study.
Recruitment	No human research participants were recruited for this study.
Ethics oversight	Ethical approval was not required for this study.

Note that full information on the approval of the study protocol must also be provided in the manuscript.

Field-specific reporting

Please select the one below that is the best fit for your research. If you are not sure, read the appropriate sections before making your selection.

☒ Life sciences ☐ Behavioural & social sciences ☐ Ecological, evolutionary & environmental sciences

For a reference copy of the document with all sections, see nature.com/documents/nr-reporting-summary-flat.pdf

Life sciences study design

All studies must disclose on these points even when the disclosure is negative.

Sample size	No calculation of sample sizes were made. We used the available datasets that have sample sizes spanning from 9 (CAMI2 Urogenital) to 1338 (HMP2).
Data exclusions	No data was excluded.
Replication	No experimental replication was performed. The method was developed based on four training datasets (MetaHit, CAMI2 Airways, Oral and Urogenital) and tested on two other datasets (CAMI2 Skin and Gastrointestinal). Further it was validated using two external datasets (Almeida et al. and Human Microbiome Project 2).
Randomization	No randomization was performed
Blinding	Investigators were not blind to the datasets, but the development of the method was done using the training datasets that were kept separate from the test datasets

Reporting for specific materials, systems and methods

We require information from authors about some types of materials, experimental systems and methods used in many studies. Here, indicate whether each material, system or method listed is relevant to your study. If you are not sure if a list item applies to your research, read the appropriate section before selecting a response.

Materials & experimental systems

n/a	Involved in the study
☒	☐ Antibodies
☒	☐ Eukaryotic cell lines
☒	☐ Palaeontology and archaeology
☒	☐ Animals and other organisms
☒	☐ Clinical data
☒	☐ Dual use research of concern
☒	☐ Plants

Methods

n/a	Involved in the study
☒	☐ ChIP-seq
☒	☐ Flow cytometry
☒	☐ MRI-based neuroimaging
